# Peripheral Arterial Disease in Diabetic Foot: One Disease with Multiple Patterns

**DOI:** 10.3390/jcm14061987

**Published:** 2025-03-14

**Authors:** Marco Meloni, Prashanth R. J. Vas

**Affiliations:** 1 Division of Endocrinology and Diabetology, Department of Medical Sciences, Fondazione Policlinico “Tor Vergata”, 00133 Rome, Italy; 2 Department of Systems Medicine, University of Rome Tor Vergata, 00133 Rome, Italy; 3 Diabetes and Diabetic Foot, King’s College NHS Foundation Trust, London SE5 9RS, UK; prashanth.vas@nhs.net; 4 Diabetes and Endocrinology, Guys and St Thomas’ NHS Foundation Trust, London SE1 9RT, UK; 5 School of Life Sciences, King’s College, London SE1 7EH, UK

**Keywords:** diabetes, diabetic foot, diabetic foot ulcers, peripheral arterial disease

## Abstract

Peripheral arterial disease (PAD) is a major complication in individuals with diabetes and is increasingly prevalent in those with diabetic foot ulcers (DFUs). Despite this, the characterisation of PAD in diabetic patients remains insufficiently refined, leading to suboptimal management and outcomes. This review underscores the necessity for a more nuanced understanding of PAD’s anatomical and biological aspects in diabetic patients. The distribution of atherosclerotic plaques varies significantly among individuals, influencing prognosis and treatment efficacy. We describe three key patterns of PAD in diabetes: pattern 1 PAD—below-the-knee (BTK) disease (with infrageniculate disease where present); pattern 2—below-the-ankle (BTA) disease; and pattern 3—small artery disease (SAD), each presenting unique challenges and require tailored therapeutic approaches. BTK PAD, characterised by occlusions in the anterior tibial, posterior tibial, and peroneal arteries, necessitates targeted revascularisation to improve foot perfusion. BTA PAD, involving the pedal and plantar arteries, is associated with higher risks of amputation and requires advanced revascularisation techniques. SAD, affecting the small arteries of the foot, remains an enigma and is challenging to treat with the current mechanical methods, highlighting the potential of autologous cell therapy as a promising alternative. A refined characterisation of PAD in diabetes is crucial for developing effective, individualised treatment strategies, ultimately improving patient outcomes, and reducing the burden of diabetic foot complications. In light of these complexities, it is incredulous that we often use a single term, “peripheral arterial disease”, to describe such a diverse array of disease patterns. This oversimplification can be perilous, as it may lead to inadequate therapeutic approaches and suboptimal patient care.

## 1. Introduction

Peripheral arterial disease (PAD) is a well-known chronic complication of diabetes, defined by the presence of atherosclerotic plaques along the peripheral vascular tree, from the sub-aortic segment to the foot. PAD is very common in persons with diabetic foot ulcers (DFUs), involving up to 50% of these patients in middle- and high-income countries [1]. The characteristics of individuals affected by DFUs have rapidly evolved in the last two decades, with a notable concomitant increase in the numbers of those affected by ischaemic DFUs compared to neuropathic DFUs [2,3]. Indeed, a recent retrospective monocentric study has documented that PAD was present in 70% of patients with a new DFU referring to a specialised diabetic foot service [4].

PAD in patients with DFU is related to worse outcomes in comparison to pure neuropathic subjects, increasing the risk of non-healing, major amputation, hospital complications and mortality [1,4]. In addition, patients with PAD usually show several attendant comorbidities, mainly cardiovascular, which influence the management and prognosis—importantly, the two comorbidities that often coexist with DFU, heart and renal failure, are independent predictors of amputation and mortality [1,4,5,6].

Even though the presence of obstructive atherosclerotic disease in the lower extremities is commonly and generally defined as PAD, the distribution of plaques could slightly or greatly vary among diabetic subjects with foot ulcers. The different distribution of arterial disease along the vascular tree is often related to the presence of some concurrent comorbidity, but the main significant data are that a different location of atherosclerotic plaques is related to a different prognosis, and the anatomy of PAD has an impact on the potential outcomes. In addition, each different pattern of PAD often needs a different treatment, including both surgical and medical approaches. Therefore, adequate study and recognition of the distribution of PAD and its biological aspect is essential to offer the best clinical approach.

In this review, we, the authors, aim to derive an overview of the main anatomical characteristics of PAD in persons with ischaemic DFUs, presenting features and discussing the different managements useful to restore foot perfusion and achieve good outcomes.

Even though the text describes the different patterns and characteristics of PAD among individuals with DFU, a section related to treating different patterns is included to help clinicians for understanding the management options. Each unique pattern requires a specific mechanical and medical strategy for improving foot perfusion and achieving the best outcomes.

Although PAD in persons with DFUs is a multisegmental disease, the authors focused their attention specifically on the main features of atherosclerotic distribution in diabetes when compared to other vascular risk factors such as smoke, hypertension, dyslipidemia, etc. Therefore, the description of arterial lesions in iliac, femoral, and popliteal districts is not reported.

In addition, the authors aim only to describe the different anatomical patterns of PAD in persons with DFUs without any interference with the classifications already present in the literature that well describe the characteristics, severity, and treatment of diabetic patients with PAD [7].

## 2. Pathophysiology in the Development of PAD

The initial pathophysiological event in the development of PAD begins with atherogenesis and advances towards atherosclerosis, ultimately impacting blood flow dynamics. Such changes can initiate early and often predate the diagnosis of diabetes [8,9,10]. This process is not confined merely to the peripheral arterial vasculature; indeed, the occurrence of polyvascular disease is common among individuals with diabetes [11,12]

**Figure 1:**
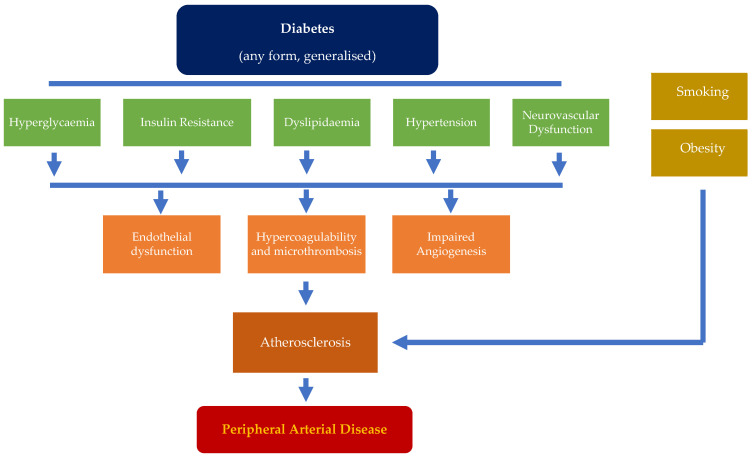
Factors influencing the development and progression of peripheral arterial disease in diabetic patients.

Many mechanisms have been identified as triggers for atherosclerosis, including hyperglycaemia [13,14,15], insulin resistance [16,17], dyslipidaemia [18,19,20], hypertension [19,20], and neurovascular dysfunction [21,22]. These can contribute individually, but perhaps more likely collectively, to stimulating endothelial dysfunction, driving hypercoagulability, micro thrombosis, and impaired angiogenesis [23,24,25]. The interplay between these factors underpins the development and progression of atherosclerosis, promoting the subsequent transformation of early atherosclerosis into stenotic/occlusive clinical PAD (Figure 1).

A highlight feature of atherosclerosis in diabetes is the presence of endothelial dysfunction leading to reduced nitric oxide (NO) synthesis. However, it is not simply unique to the condition, being present in multiple conditions which are often coexistent, such as hypertension and dyslipidaemia. Atherogenesis leads to the subendothelial accumulation of lipid-rich monocyte-derived foam cells and associated T cells, which coalesce to form a non-stenotic fatty streak [26]. As the deposition progresses, a necrotic core of cholesterol esters is formed, covered by a fibrous cap containing vascular smooth muscle cells (VSMC) and inflammatory cells (T cells, mast cells, and some macrophages). Evidence has shown abnormal neovascularisation around atherosclerotic lesions and calcium hydroxyapatite deposition [16,26].

Chronic hyperglycaemia may also drive the production of advanced glycation end products (AGEs) [27], activating protein kinase C, which downstream activates multiple pro-inflammatory genes. This can reduce NO synthesis and further impair vasodilation. Insulin resistance can also directly impair vascular endothelial function by disturbing subcellular signalling pathways common to insulin action and nitrate oxide production.

Diabetic vascular smooth muscle cells also exhibit a higher predisposition to undergo osteogenic transformation [28,29]. The increased calcium and phosphate deposition stiffens the arterial walls. The end result, medial arterial calcification (Mönckeberg sclerosis), is a common finding in individuals with diabetes, reducing vessel wall compliance [30,31]. The presence of autonomic neuropathy in diabetes can affect blood vessel wall blood flow regulation. Impaired sympathetic control has its own potential to reduce vasodilation, thereby exacerbating the ischaemic effects [32].

Moreover, exogenous factors such as obesity and smoking, which often coexist, can trigger a cascade of independent mechanisms that lead to the formation of atherosclerotic plaques. The interaction of harmful toxins released in cigarette smoke, a genetic predisposition, and the influence of other risk factors in an individual with diabetes can further accelerate the manifestation of peripheral arterial disease [33]. Abnormal catecholamine release stimulates the endothelial ‘alpha-adrenergic’ receptors and diminishes nitric oxide availability in these cells.

While atherosclerosis was once considered a degenerative, progressive condition, recent evidence indicates that many atherosclerotic regions are actually supported by a dynamic inflammatory process that can be modified [26]. Supporting this, trials of lipid-lowering agents (e.g., statins) and more recent antidiabetic medications (e.g., SGLT2 inhibitors and GLP-1 analogues) designed for anti-hyperglycaemic purposes have demonstrated improved clinical outcomes for individuals with peripheral arterial disease [34,35]. 

## 3. Common Characteristics and Anatomical Distribution of PAD in Persons with Diabetic Foot

**Figure 2:**
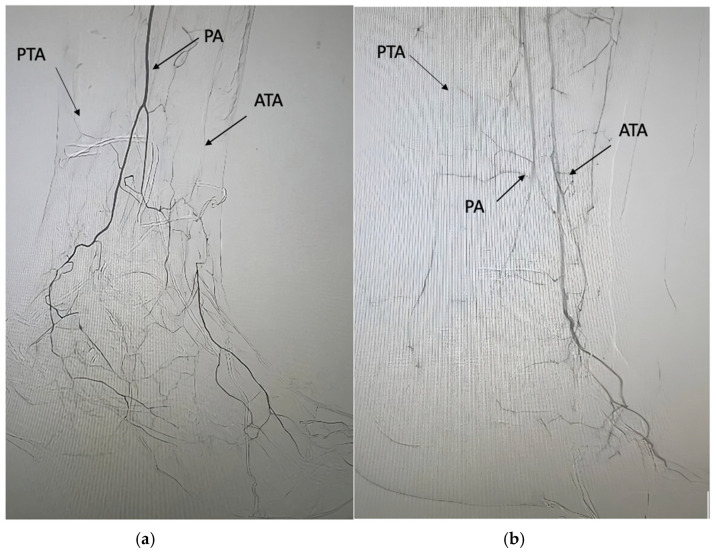
(**a**). Arterial disease with occlusion of anterior tibial (ATA) and posterior tibial artery (PTA). Foot perfusion is partially ensured by the peroneal artery (PA). (**b**). Arterial disease with occlusion of the posterior tibial artery (PTA) and stenosis of the distal part of the peroneal artery (PA). Foot perfusion is partially ensured by the anterior tibial artery (ATA) presenting some distal stenosis.

PAD in persons with diabetes is defined by the presence of obstructive atherosclerotic disease in lower extremities arteries. PAD in diabetics affects younger subjects, shows a multisegmental distribution, and a faster progression in comparison to persons without diabetes [36,37]. The arterial vessels are often characterised by widespread calcification, and occlusions are more frequent than stenosis [36,37,38]. Diabetes affects neoangiogenesis, and the presence of collateral vessels is often reduced [36,37]. Even though PAD in diabetic persons shows a multi-level distribution from the iliac segments to the foot, the distribution of atherosclerotic plaques and arterial lesions in diabetic subjects is greatly characterised by the involvement of below-the-knee (BTK) arteries (anterior tibial artery, posterior tibial artery, and peroneal artery) when compared to PAD in persons without diabetes [39,40,41]. Based on the aim of this review, this kind of PAD with the involvement of BTK arteries (with or without the involvement of iliac, femoral, and popliteal arteries) will be defined in the text as pattern 1 (Table 1, Figure 2a,b).

The involvement of infra-popliteal vessels in diabetes has been associated with a higher risk of major amputation in comparison to PAD exclusively characterised by the involvement of above-the-knee (ATK) arteries [39].

## 4. Evolution and New Pattern of PAD in Patients with Diabetic Foot Ulcers

**Figure 3:**
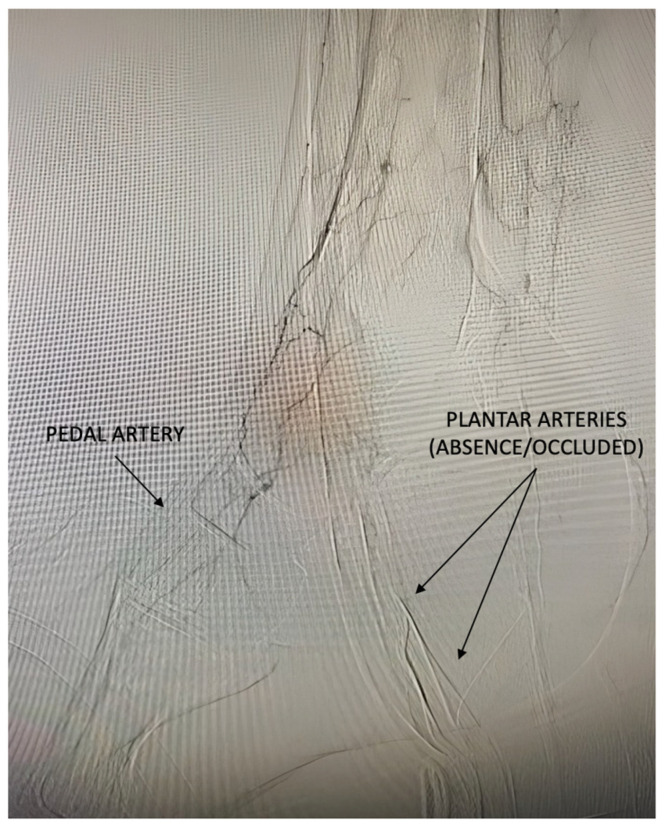
Below-the-ankle arterial disease with the absence/occlusion of the pedal and plantar arteries in a patient presenting an open tibial artery.

While the involvement of infra-popliteal vessels in diabetes is a well-established concept, the pattern of PAD in persons with diabetes and ischaemic foot ulcers appears to include certain distinct and more aggressive forms. In recent years, it has been documented that PAD can often present more distally, profoundly involving the foot arteries that were previously under-considered in diabetic subjects [42]. Foot arteries, such as the dorsalis pedal artery, medial plantar artery, and lateral plantar artery, are now recognised as major arteries of the foot, primarily responsible for delivering blood to various areas of the foot [42]. Authors consider this kind of PAD a severe pattern of PAD, and it will be defined in the text as pattern 2 of PAD (Table 1, Figure 3).

In a very large retrospective study, Ferraresi et al. documented that among 1915 angiograms of patients affected by chronic limb-threatening ischaemia (CLTI), approximately 80% of them were affected by the arterial disease of foot arteries [42]. The presence of below-the-ankle [BTA] arterial disease was found to be the more aggressive clinical pattern of CLTI, being associated with foot gangrene and increased risk of amputation. Furthermore, diabetes and end-stage renal disease (ESRD) emerged as significant factors associated with BTA arterial disease [42]. Another recent study focusing on individuals with ischaemic DFUs analysed the prevalence of BTA disease and its relationship with clinical outcomes [43]. Among 272 patients with ischaemic DFUs who underwent lower limb revascularisation, BTA arterial disease was retrospectively identified in 44% of the cases. Patients with BTA arterial disease exhibited higher rates of cardiovascular risk factors such as ischaemic heart disease, ESRD, and hypertension. Notably, individuals with BTA involvement experienced higher rates of non-healing, minor amputation, major amputation, mortality, and revascularisation failure compared to those without BTA involvement [43]. Additionally, the presence of BTA arterial disease was found to be an independent predictor of non-healing and secondary minor amputation [43].

While PAD in diabetes is generally associated with atherosclerotic disease in other vascular districts, this specific pattern of PAD involving foot arteries appears to be more closely associated with the presence of CAD. One recent study compared the prevalence of CAD in patients with ischaemic DFUs presenting with BTK disease without BTA involvement and those with BTA arterial disease. The results indicated that the presence of foot artery disease was more strongly associated with CAD [44]. In this study, the overall prevalence of CAD in the entire population was 63.4%. However, within the BTA group, the prevalence was significantly higher at 75.4%, compared to 54.1% in the BTK group [44]. Patients with BTA arterial disease reported more cases of acute myocardial infarction (AMI) (5%) during 1 year of follow-up when compared to BTK patients in which AMI occurred in 1.3% of the cases. In addition, BTA arterial disease (compared to BTK arterial disease) was independently associated with the presence of CAD [44]

We believe this reinforces the need for screening CAD in all individuals with DFUs and PAD, suggesting a much lower threshold for investigation in those with foot artery disease.

## 5. The Small Artery Disease: A Mysterious Pattern of PAD

**Figure 4:**
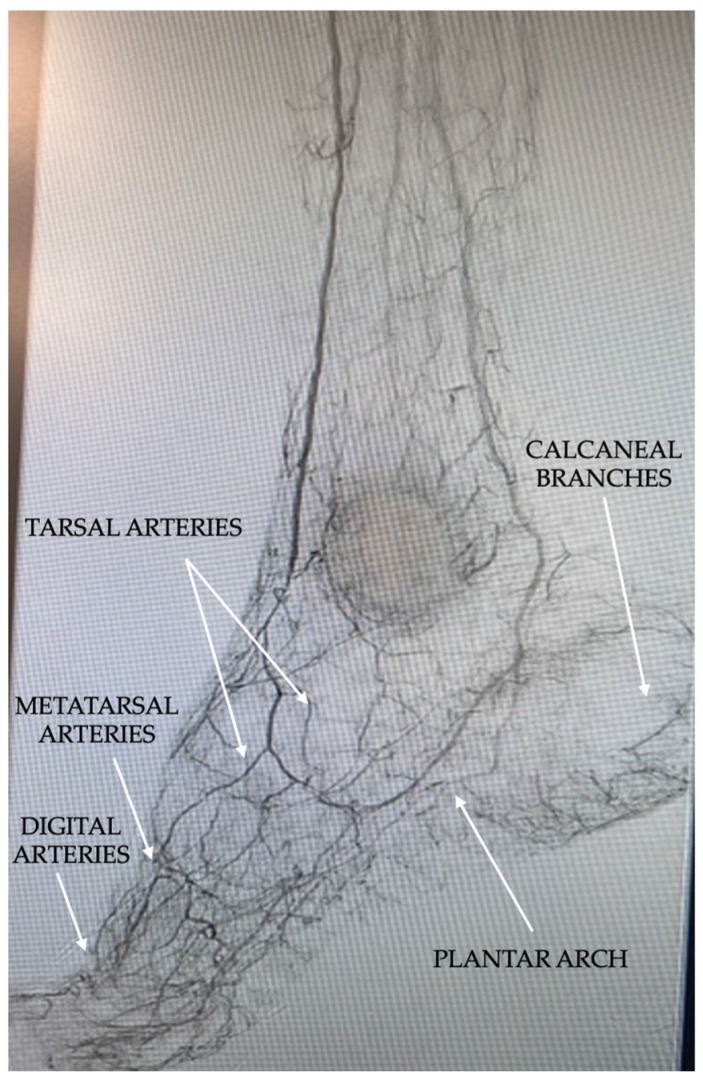
Normal distribution of small arteries into the foot.

The major foot arteries, including the dorsalis pedis and the lateral and medial plantar arteries, are responsible for blood transmission to the foot. In contrast, the smaller arteries, which are referred to as the small vessels of the foot, facilitate blood distribution into the foot tissue. The small arteries of the foot comprise the plantar arch and the arteries originating from it such as the tarsal arteries, metatarsal arteries, and digital arteries, but also the calcaneal branches of the plantar arteries (Figure 4).

**Figure 5:**
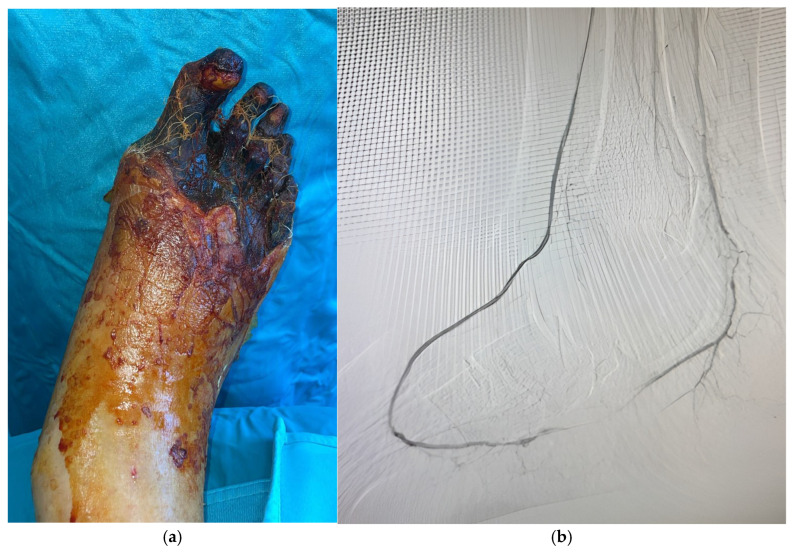
(**a**) Gangrene of forefoot in a patient with small artery disease (grade 3) with preserved major foot arteries after revascularisation procedure (**b**). Despite revascularisation, there is an absence of visible flow into the digital and metatarsal arteries. (**b**).

Small artery disease (SAD) is, therefore, an arterial disease involving the above-mentioned arteries. SAD is confirmed by a global angiographic evaluation of the plantar arch and small arteries. SAD can be present in different grades of severity: (1) patent with the absence of disease or mild disease with a preserved network of the forefoot and calcaneal branches; (2) diffuse disease with the narrowing or poverty of digital, metatarsal, tarsal, and calcaneal branches; (3) extreme poverty or absence of plantar arch, digital, metatarsal, tarsal, and calcaneal branches. The authors consider this kind of PAD as an extremely severe pattern of PAD, and it will be defined as pattern 3 of PAD (Table 1, Figure 5).

It is estimated that up to 25% of individuals with diabetes and PAD demonstrate the presence of SAD, which often coexists with BTA disease [42,45]. Diabetes is a powerful risk factor for SAD (relative risk > 2 times in comparison to the absence of diabetes), while other factors associated with the presence of SAD are ESRD and obesity [42].

Even though the definition of SAD could be not so precise and requires specialised expertise for its evaluation, it seems to play a crucial role in the scenario of PAD, resulting in being independently associated with CLTI which represents the most severe clinical pattern of PAD [42]. Even though there is a paucity of clinical studies, the presence of SAD could play a significant role in tissue lesions.

## 6. Diagnostic Assessment

The detection of PAD typically involves a sequence of diagnostic steps. Firstly, a broad clinical evaluation is required to identify potential risk factors for lower limb atherosclerotic disease, including age, diabetes duration, obesity, hypertension, dyslipidemia, ESRD, and smoking. For instance, the presence of ESRD could indicate the potential presence of BTA arterial disease and SAD [42], while obesity seems to be related to SAD [42].

Clinical history should be followed by the identification of peripheral pulses (femoral, popliteal, dorsalis pedal, and posterior tibial artery): the absence of pulses could be related to the presence of PAD in different levels of the vascular tree, while their presence cannot completely exclude the presence of PAD, especially in the case of several cardiovascular risk factors and/or the presence of signs of ischaemic wounds such as necrosis or gangrene [46,47].

The diagnosis of PAD is ultimately determined through a comprehensive set of laboratory tests. Diagnostic assessment includes first-level tests such as ankle-brachial index (ABI), ankle pressure (AP), toe-brachial index (TBI), toe pressure (TP), transcutaneous oxygen pressure (TcPO2), and ultrasound (US) colour duplex, while magnetic resonance imaging (MRI) and computed tomography (CT) are defined as second-level tests [7].

PAD is highly suspected in subjects with DFUs presenting ABI < 0.9 or >1.3 and TBI < 0.70 [47]. Normal ABI values are considered between 0.9 and 1.3, and in the case of incompressible arteries due to the presence of calcifications, ABI values could be falsely normal or higher in patients with DFUs [48]. AP and ABI are also considered weak predictors of healing: AP < 50 mmHg or ABI < 0.5 could be associated with reduced chances of healing and increased risk of major amputation [49].

TP and TBI can assess the distal blood flow, specifically in the forefoot and toes [50]. They also predict the potential probability of wound healing: TP values > 30 mmHg increases the chance of healing up to 30%; otherwise, TP values < 30 mmHg increase the risk of major amputation nearly to 20% [51].

TcPO2 is a useful tool for identifying a PAD condition and its grade of severity. In addition, TcPO2 is usually considered in the clinical practice to predict the chance of healing in patients with DFUs [7]. TcPO2 values between 30 and 50 mmHg are suggestive of mild/moderate PAD, while TcPO2 values < 30 mmHg are considered a condition of CLTI and, in this case, lower limb revascularisation should be mandatory [52].

In addition, TcPO2 values > 50 mmHg are a good predictor of wound healing in patients with DFUs, while values < 25 mmHg are suggestive of non-healing [7]. The chances of healing should be considered uncertain in the case of values between 25 and 50 mmHg, and additional variables of the wound, such as the size, depth, and presence of infection, should be taken into account before considering revascularisation options [7,53].

TcPO2 is also a reliable tool for evaluating the wound angiosome blood perfusion and can be tested in different areas of the foot according to the ulcer location [54]. Accordingly, TcPO2 could help clinicians decide whether they should perform a revascularisation procedure. Different conditions could interfere with the sensitivity of TcPO2, mainly peripheral oedema and infection, two conditions that can reduce oxygen values [54]. TcPO2 is not able to define the location of atherosclerotic disease in the vascular tree but it can greatly help clinicians to define the grade of blood supply in the wound angiosome area in the case of incomplete revascularisation (BTK or BTA). In the case of persistent ischaemia in the wound angiosome area, a new revascularisation (if technically feasible) or the use of some different adjuvant therapies could be considered [55]. The same concept could be evaluated in the case of SAD. If, in the case of SAD, TcPO2 identifies the persistence of foot/angiosome ischemia, the use of new regenerative therapies should be closely considered due to its effectiveness in modifying the vascular condition in the case of SAD, by increasing TcPO2 levels and promoting wound healing [55,56].

Although the test are reliable and accurate in a large part of the population, there is a potential risk of several errors, which could influence the sensitivity and specificity of each functional assessment. Therefore, the above-mentioned bedside tests should be performed by trained health care professionals in a standardised protocol [47]. In addition, even though they can identify the potential presence of PAD (individually or combined), these tests do not indicate the site of atherosclerotic disease in the vascular tree.

US colour duplex is a very good tool to define the anatomical distribution of atherosclerotic plaques across the vascular tree and its impact on foot perfusion [7,57]. When expert vascular professionals perform the analysis, the information acquired can locate the stenotic/occlusive PAD region, reliably identifying the presence of BTK and/or BTA arterial disease. Nonetheless, the same exam is not useful for screening the presence of SAD. US colour duplex, in association with clinical parameters, can help clinicians to define the need for revascularisation procedure and map the type of approach and which vessel deserves recanalisation or not [56,57].

MRI and CT are the gold standard for studying and describing PAD. Both provide a clear framework of arterial lesions and their distribution. They can often better define the presence of BTK and BTA arterial disease and, in some cases, detect the presence of SAD. Their use can help vascular surgeons or interventional radiologists/cardiologists in some specific circumstances to plan the vascular approach, especially in the case of some doubt on US evaluation, atherosclerotic disease involving iliac arteries or common femoral artery, and previous vascular interventions (especially bypass [7,58,59]).

Angiography should not be commonly used as a diagnostic test, but only when a revascularisation procedure by endovascular approach is already planned [7,60]. In this specific case, it can be considered the final diagnostic step just before the revascularisation procedure, allowing one to better identify the type of PAD (BTK and/or BTA), the precise location of atherosclerotic plaques, the characteristics of plaques, and finally, the presence of SAD [42,60].

## 7. Treatment

In persons with ischaemic DFUs and PAD who are suitable for peripheral revascularisation, it is essential to evaluate the entire lower extremity arterial circulation (from iliac segments to the foot) with detailed visualisation of BTK and BTA arteries [7]. The aim of revascularisation procedures should be to restore in-line blood flow to at least one of the foot arteries [46]. Recovering the optimal blood flow increases the chance of healing and reduces the risk of amputation. Otherwise, incomplete revascularisation can impact the chance of wound healing and healing time [7].

Basically, revascularisation should be addressed to recanalise the wound-related artery which supplies the anatomical region of the ulcer when this approach is feasible [7]. In the case of ischaemic DFUs requiring restoration of blood perfusion, early revascularisation (preferably within two weeks from the assessment) is essential to achieve the best outcomes [61,62].

Nonetheless, revascularisation procedures should be performed according to the characteristics of PAD, considering the anatomical distribution of atherosclerotic plaques and the wound location. According to the preliminary assessment of PAD, revascularisation procedures (both surgical bypass and endovascular) should aim to recanalise all the stenotic/occluded arteries that could influence the foot perfusion, including also iliac, femoral, and popliteal arteries [7]. Even though iliac arteries and ATK arteries are not deeply described in the current review based on the aim of the authors, the mentioned vessels deserve the same careful consideration and need to be recanalised in the case of significant stenosis and occlusion, regardless of the baseline angiographic pattern [63,64]

It is established that PAD in diabetes is a multi-level disease; until a few years ago, the main severe pattern of PAD was considered the pattern 1 involving BTK arteries, and the revascularisation was targeted to recanalise infra-popliteal vessels. As reported in a large study by Faglia et al. [65], the recanalisation of at least a vessel BTK (mainly anterior tibial arteries, and as a second option the peroneal artery) was mandatory to achieve the recovery of blood perfusion into the foot tissues and promote wound healing. Opening one BTK vessel more seemed to ensure better results than the revascularisation of a single BTK vessel [65]. This result can be achieved regardless of the kind of approach, surgically (open bypass) or endovascular (percutaneous transluminal angioplasty).

Nonetheless, considering the high prevalence of BTA arterial disease, nowadays in several cases, the recanalisation of BTK vessels is not sufficient to ensure good outcomes. Even though Huzing et al. did not find any significant difference in limb salvage and amputation-free survival between BTA and BTK angioplasty [66], the role and the management of BTA arterial disease remains an open debate. More recently, it has been documented that in the case of BTA arterial disease, the revascularisation of foot arteries is crucial for achieving wound healing and avoiding major amputation [41,67]. In a recent study on this specific topic, patients with BTA arterial disease receiving a complete recanalisation of foot arteries allowed up to 89% of healing and only 2.1% of major amputation at 1 year of follow-up, while a failed foot revascularisation led to less chance of healing (9.1%) and higher risk of major amputation (36.3%) [67]. In addition, failed foot revascularisation is an independent predictor of non-healing, minor amputation, and major amputation.

Given the increased prevalence of BTA arterial disease in patients with DFUs and its significant impact on wound healing, several advanced vascular techniques have been developed to recanalise foot arteries. These techniques include plantar-loop techniques, retrograde revascularisation, and bifurcated bypass, among others [68,69,70,71], which appear to yield better results, particularly when standard revascularisation fails. However, the revascularisation of BTA arteries is not always feasible and proves to be more challenging than BTK revascularisation, with a consistent rate of revascularisation failure [72,73,74].

**Figure 6:**
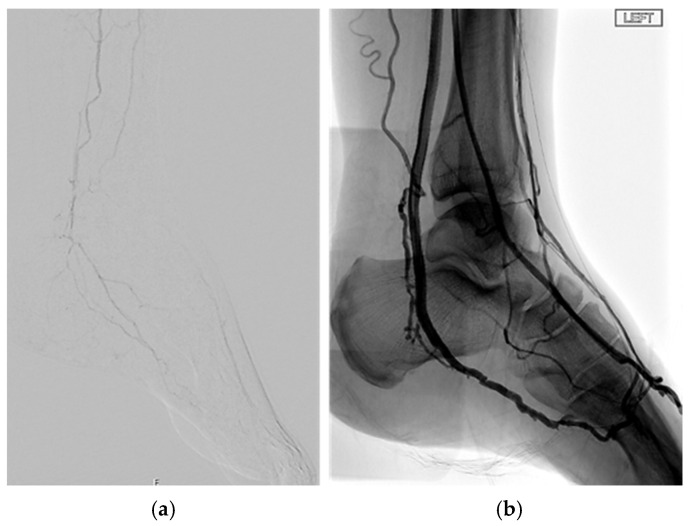
Presence of pattern 2 and pattern 3 PAD in an individual with diabetes (**a**) treated with percutaneous deep venous arterialisation (**b**).

More recently, an endovascular or percutaneous deep venous arterialisation (pDVA) has been developed, wherein anastomosis of arterial inflow and venous outflow permits oxygenated blood to enter the veins and perfuse devitalised tissues via the retrograde route [75,76] (Figure 6).

A recent systematic review of 233 participants with no-option ischemia undergoing pDVA [77] reported a technical success rate of 97% (95% confidence interval [CI] 96.2–97.9%) and a major amputation rate of 21.8% (95% CI 21.1–22.4%) at one year. The rate of wound healing was 69.5% (95% CI 67.9–71.0%), the reintervention rate was 37.4% (95% CI 34.9–39.9%), and the all-cause mortality rate was 15.7% (95% CI 14.1–17.2%).

Even though peripheral ischaemia and its characteristics severely impact the prognosis of patients with DFUs, the presence of other significant variables such as infection, depth (bone involvement), size, inability to stand or walk without help (frailty), dialysis, and heart failure could reduce the probability of wound healing and should be always considered [1,4,6].

While BTK arteries and major BTA arteries can potentially be approached mechanically through a bypass procedure or an endovascular technique, the mechanical treatment of small arteries remains hugely challenging due to their anatomy and distribution. Therefore, SAD is often a real burden in revascularisation strategies, even in the case of the successful recanalisation of the larger foot arteries.

SAD has often been found in patients with no-option limb ischaemia who presented a failure of mechanical revascularisation procedure [41,44] or in those patients who receive indirect revascularisation and show the persistence of wound angiosome ischaemia despite the successful recanalisation of one of the foot arteries [78,79,80,81]. In the first mentioned framework, SAD is usually associated with the absence of big foot arteries (pedal artery and plantar arteries) defining the so-termed condition of “desert foot” in which there is a complete or almost absence of blood flow below the ankle. This condition, without recourse to rescue treatment, is associated with the highest risk of major amputation [81,82].

The second framework is otherwise related to a partial recanalisation of the foot but not of the wound-related artery (indirect revascularisation). In the case of SAD, there are no active collateral vessels that can supply the absence of direct flow to the wound angiosome area which could remain in a persistent or partially ischaemic status. The absence of viable collateral vessels increases the risk of non-healing, longer healing time, and higher risk of amputation when compared to indirect revascularisation with the presence of good collateral vessels [78,79,80,81,82,83].

Until a few years ago, the treatment of conditions such as desert foot or indirect revascularisation was considered not feasible and often, major amputation was the clinical choice. In recent years, new frontiers for treating these severe frameworks of PAD have been opened offering new solutions for clinicians involved in the field.

Particularly, the use of autologous cell therapy (ACT), mainly mononuclear cell therapy collected from the peripheral blood (PB-MNC) or from the bone marrow (BM-MNC), seemed to change the natural history of untreatable limb ischaemia in persons with DFUs [84]. Even though more consistent studies and the largest clinical trials are required, ACT has been documented to improve foot ischaemia, reduce foot ischaemic pain, increase the chance of healing and limb salvage, and reduce mortality [84,85,86,87]. Scatena et al. have documented that ACT was associated with a significantly higher rate of ulcer healing (>80%), greater increase of TcPO_2_ and ABI values, and reduction in pain at 2 years of follow-up in comparison to conventional therapy in persons with DFUs and no-option CLTI [88]. Panunzi et al. have documented similar clinical results in a single-arm study documenting that ACT was effective in the case of SAD by the increase in the local factors involved in the paracrine neoangiogenesis [89]. Monami et al. showed a positive impact on limb salvage and improvement in ischaemic parameters (TcPO2) by the use of PB-MNCs in diabetic patients with no-option CLTI and SAD. The same study recorded 20% of major amputations at 1-year follow-up and amputees were those presenting the most severe pattern of SAD with the complete absence of small arteries [56].

Meloni et al. documented a reduction of major amputaton nearly to 50% at 1-year of follow-up in patients with DFUs and NO-CLI by using PB-MNCs in comparison to patients treated by conventional therapy [90]. Afterwards, the same research group documented the effectiveness of PB-MNC therapy in patients with DFUs and SAD receiving indirect revascularisation with the persistence of wound angiosome ischaemia. At 1-year of follow-up, wound healing was achieved in more than 80% of cases and any amputation was recorded [55]

**Figure 7:**
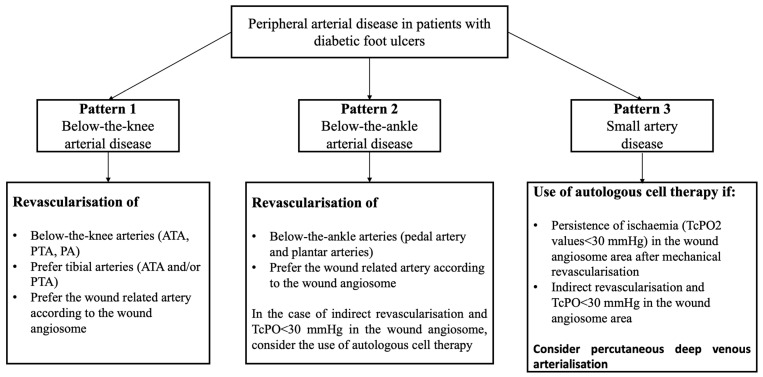
Suggested algorithm of treatment according to the different patterns of PAD.

An easy algorithm reported in Figure 7 suggests a summary of the treatment of the three different patterns.

## 8. Early Detection of PAD and Global Management

Early diagnosis of PAD is mandatory for improving outcomes in terms of healing and limb salvage, but also to reduce cardiovascular morbidity and mortality [90]. The assessment of foot blood perfusion should be ensured being PAD one of the main reasons for non-healing or delayed healing in persons with DFU [1]. When PAD is detected and blood flow to the foot is not sufficient for wound healing, early revascularisation should be performed to improve outcomes [61,62].

PAD is a well-known severe risk factor for cardiovascular events [35], and the global management of all cardiovascular aspects should be required, including glycemic control, smoking, lipids, and blood pressure. Glycemic control should be firstly considered to reduce the progression of atherosclerotic disease over time [46,91]. In addition, impaired control of diabetes could negatively impact the wound-healing process and limb salvage [92], as well as mortality in admitted patients [93]. Usually, HbA1c values < 7.0% should be ensured with the goal of reducing the progression of lower limb atherosclerotic disease, even if this target needs to be adapted according to any individual condition [93].

Smoking cessation is also mandatory to reduce the worsening of PAD and the risk of any related cardiovascular events [94]. In addition, smoking is associated with a high risk of revascularisation failure and the recurrence of limb ischaemia post-revascularisation [95,96].

Dyslipidaemia, including total cholesterol, low-density lipoprotein cholesterol (LDL-C), triglycerides, and lipoprotein a, is closely associated with PAD and the promotion of atherosclerotic disease in several vascular districts [97]. In patients with PAD and those managed by lower limb revascularisation procedures, the use of statin therapy is widely recommended [94,98].

At the same time, the adequate management of blood pressure should be ensured to further reduce the progression of PAD. Usually, in patients with diabetes pressure values < 130/80 mmHg are the target required, but in older persons, higher values are recommended for avoiding severe hypotension events [94,99].

Any available drug for reducing hypertension could be used, even though in patients with diabetes, angiotensin-converting enzyme (ACE) and angiotensin receptor are considered the first-line treatment for their positive effects on cardiovascular morbidity and events [95,100]. In addition, the use of ACE and/or ARB has documented a reduction in long-term mortality in patients affected by DFUs [100].

Diabetic foot syndrome is considered a complex disease and technical vascular aspects are only an element to be considered. All cardiovascular risk factors, such as screening and education of patients in their personal foot care, deserve the same attention to reduce the risk of impairment of both PAD and foot ulcers [7]. Accordingly, global management by a multi-disciplinary team is recommended for improving outcomes also in ischaemic subjects [101].

## 9. Conclusions

PAD in patients with ischaemic diabetic foot is not a single disease, showing three main patterns defined by the involvement of BTK arteries, BTA arteries, and small foot arteries, respectively. Each pattern has a different severity, with BTA arterial disease and SAD presenting the highest risk of being unsuccessful in their respective treatment. While BTK arterial disease needs the recanalisation of infra-popliteal vessels, preferably of the artery direct to the wound angiosome area, to achieve wound healing and avoid major amputation, BTA arterial disease requires the recanalisation of foot big arteries (pedal and plantar arteries). SAD is not still treatable by revascularisation procedures but seems to improve using ACT, specifically mononuclear cell therapy, which ensures the creation of small new vessels in the area of ischaemia.

Further studies will be useful to better define the diagnosis of SAD by non-invasive tools (nowadays evaluable only by the angiographic evaluation) and identify the response to each medical approach to decide what could be the best management of ischaemic DFUs.

As reported in the introduction, the authors aim only to describe the different anatomical patterns of PAD in persons with DFUs without underestimating or substituting the classifications on the severity of PAD in diabetic patients and its treatment widely and well reported by the recent guidelines of IWGDF.

The authors state that the patterns described in the text are related to the extension of PAD and merely report their point of view based on the angiographic distribution of arterial lesions in patients with DFUs. Outcomes related to the different patterns represent data collected by a few papers available in the literature, and the majority of them are retrospective studies with different kinds of populations. This point could be a bias and other research could be useful to reinforce this kind of description.

## Figures and Tables

**Table 1:** Pattern of peripheral arterial disease in patients with ischaemic diabetic foot ulcers.

Pattern 1	Pattern 2	Pattern 3
Presence of below-the-knee arterial disease (anterior tibial artery, posterior tibial artery, and peroneal artery) Presence or absence of above-the-knee arterial disease (iliac arteries, femoral arteries, popliteal artery, and tibio-peroneal trunk) Absence of below-the-ankle arterial disease	Presence of below-the-ankle arterial disease (pedal artery, medial plantar artery, and lateral plantar artery) Presence or absence of below-the-knee and above-the-knee arterial disease Absence of small arterial disease	Presence of small artery disease (plantar arch, digital, metatarsal, tarsal, and calcaneal branches) Presence or absence of below-the-ankle and below-the-knee arterial disease
